# Native myocardial T1 and right ventricular size by CMR predict outcome in systemic sclerosis-associated pulmonary hypertension

**DOI:** 10.1093/rheumatology/keae141

**Published:** 2024-05-17

**Authors:** Daniel S Knight, Ruta Virsinskaite, Nina Karia, Alice R Cole, Rory H Maclean, James T Brown, Rishi K Patel, Yousuf Razvi, Lucia Venneri, Tushar Kotecha, Ana Martinez-Naharro, Peter Kellman, Ann M Scott-Russell, Benjamin E Schreiber, Voon H Ong, Christopher P Denton, Marianna Fontana, J Gerry Coghlan, Vivek Muthurangu

**Affiliations:** National Pulmonary Hypertension Service, Royal Free London NHS Foundation Trust, London, UK; Department of Cardiac MRI, Royal Free London NHS Foundation Trust, London, UK; Institute of Cardiovascular Science, University College London, London, UK; National Pulmonary Hypertension Service, Royal Free London NHS Foundation Trust, London, UK; Department of Cardiac MRI, Royal Free London NHS Foundation Trust, London, UK; Institute of Cardiovascular Science, University College London, London, UK; National Pulmonary Hypertension Service, Royal Free London NHS Foundation Trust, London, UK; Institute of Cardiovascular Science, University College London, London, UK; Centre for Rheumatology and Connective Tissue Diseases, UCL Medical School (Royal Free Campus), London, UK; Centre for Rheumatology and Connective Tissue Diseases, UCL Medical School (Royal Free Campus), London, UK; National Pulmonary Hypertension Service, Royal Free London NHS Foundation Trust, London, UK; Department of Cardiac MRI, Royal Free London NHS Foundation Trust, London, UK; Institute of Cardiovascular Science, University College London, London, UK; Department of Cardiac MRI, Royal Free London NHS Foundation Trust, London, UK; Division of Medicine, University College London, London, UK; Department of Cardiac MRI, Royal Free London NHS Foundation Trust, London, UK; Division of Medicine, University College London, London, UK; Department of Cardiac MRI, Royal Free London NHS Foundation Trust, London, UK; National Pulmonary Hypertension Service, Royal Free London NHS Foundation Trust, London, UK; Department of Cardiac MRI, Royal Free London NHS Foundation Trust, London, UK; Institute of Cardiovascular Science, University College London, London, UK; Department of Cardiac MRI, Royal Free London NHS Foundation Trust, London, UK; National Heart, Lung, and Blood Institute, National Institute of Health, Bethesda, MD, USA; Department of Rheumatology, Queen Alexandra Hospital, Portsmouth, UK; National Pulmonary Hypertension Service, Royal Free London NHS Foundation Trust, London, UK; Centre for Rheumatology and Connective Tissue Diseases, UCL Medical School (Royal Free Campus), London, UK; Centre for Rheumatology and Connective Tissue Diseases, UCL Medical School (Royal Free Campus), London, UK; Department of Cardiac MRI, Royal Free London NHS Foundation Trust, London, UK; Division of Medicine, University College London, London, UK; National Pulmonary Hypertension Service, Royal Free London NHS Foundation Trust, London, UK; Institute of Cardiovascular Science, University College London, London, UK

**Keywords:** CMR, T1 mapping, prognosis, pulmonary hypertension, right ventricle, systemic sclerosis

## Abstract

**Objectives:**

Measures of right heart size and function are prognostic in systemic sclerosis-associated pulmonary hypertension (SSc-PH), but the importance of myocardial tissue characterisation remains unclear. We aimed to investigate the predictive potential and interaction of cardiovascular magnetic resonance (CMR) myocardial tissue characterisation and right heart size and function in SSc-PH.

**Methods:**

A retrospective, single-centre, observational study of 148 SSc-PH patients confirmed by right heart catheterization who underwent clinically indicated CMR including native myocardial T1 and T2 mapping from 2016 to 2023 was performed.

**Results:**

Sixty-six (45%) patients died during follow-up (median 3.5 years, range 0.1–7.3). Patients who died were older (65 *vs* 60 years, *P* = 0.035) with more dilated (*P* < 0.001), hypertrophied (*P* = 0.013) and impaired (*P* < 0.001) right ventricles, more dilated right atria (*P* = 0.043) and higher native myocardial T1 (*P* < 0.001).

After adjustment for age, indexed right ventricular end-systolic volume (RVESVi, *P* = 0.0023) and native T1 (*P* = 0.0024) were independent predictors of all-cause mortality. Both RVESVi and native T1 remained independently predictive after adjusting for age and PH subtype (RVESVi *P* < 0.001, T1 *P* = 0.0056). Optimal prognostic thresholds for RVESVi and native T1 were ≤38 mL/m^2^ and ≤1119 ms, respectively (*P* < 0.001). Patients with RVESVi ≤ 38 mL/m^2^ and native T1 ≤ 1119 ms had significantly better outcomes than all other combinations (*P* < 0.001). Furthermore, patients with RVESVi > 38mL/m^2^ and native T1 ≤ 1119 ms had significantly better survival than patients with RVESVi > 38mL/m^2^ and native T1 > 1119ms (*P* = 0.017).

**Conclusion:**

We identified prognostically relevant CMR metrics and thresholds for patients with SSc-PH. Assessing myocardial tissue characterisation alongside right ventricular function confers added value in SSc-PH and may represent an additional treatment target.

## Graphical Abstract

**Figure:**
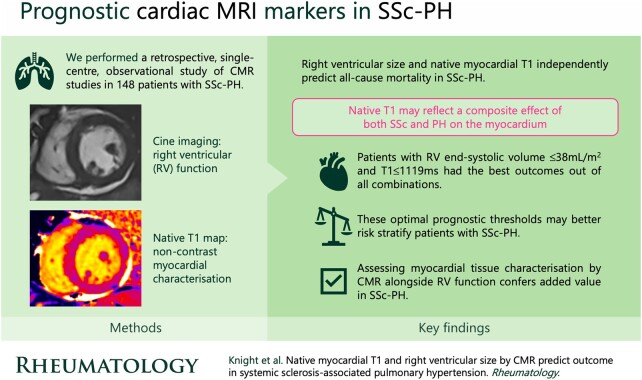

Rheumatology key messages

- Right ventricular size and native myocardial T1 by CMR independently predict all-cause mortality in SSc-PH.
- Prognostic thresholds of these CMR metrics may better risk stratify patients with SSc-PH.
- Assessing myocardial tissue characterisation by CMR alongside RV function confers added value in SSc-PH.

## Introduction

Pulmonary hypertension (PH) is a recognized complication of systemic sclerosis (SSc), with aetiologies including pulmonary arterial hypertension (PAH, group 1 PH), left heart disease (group 2) and lung disease (group 3) [1]. Common to all PH subtypes complicating SSc are increased morbidity and mortality, hence the need to develop better biomarkers that predict outcomes in SSc-PH. In idiopathic PAH (IPAH), measurements of right heart size and function by cardiovascular magnetic resonance (CMR) are recognized prognostic markers [2, 3]. However, patients with SSc-PH may also have additional and unrelated cardiovascular issues that may affect outcome. In particular, myocardial fibrosis and oedema, as reflected by elevated native T1 on CMR, are known prognostic findings in patients with SSc [4]. Yet, it is not known if myocardial tissue characterisation provides additional predictive information in SSc-PH over and above right heart size.

The aims of this study were to (i) investigate the predictive potential and interaction of myocardial tissue characterisation and right heart size and function in patients with SSc-PH and (ii) determine useful thresholds of prognostic metrics that could be used in clinical practice.

## Methods

### Patient population

We performed a retrospective, single-centre, observational study of 148 consecutive patients with SSc-PH who underwent CMR at the Royal Free Hospital, London, United Kingdom (UK) from 27^th^ January 2016 to 29^th^ March 2023. One hundred ten patients with complete CMR datasets were included in a previous analysis of 260 patients with SSc (with or without PH) as a common diagnosis [4]. Pulmonary hypertension was confirmed in all patients by right heart catheterisation as mean pulmonary arterial pressure (mPAP) ≥25 mmHg, with the PH subtype diagnosed by the clinical team according to the clinical classification of PH, specifically: group 1, pulmonary arterial hypertension; group 2, PH due to left heart disease (pulmonary arterial wedge pressure, PAWP, >15 mmHg); group 3, PH due to lung diseases and/or hypoxia; and group 4, chronic thromboembolic PH [5]. All patients, including those with a concomitant overlap syndrome diagnosis, fulfilled the American College of Rheumatology/European League Against Rheumatism 2013 classification criteria for SSc [6]. Patient clinical histories, co-morbidities and haemodynamic data were obtained from patient electronic records systems and specialist services databases by clinicians blinded to the patient outcomes. A validated SSc disease severity score at the time of the scan from 0 (no documented involvement) to 4 (end-stage disease) was derived as described previously [7]. The outcome was ascertained by checking the patient summary care record on the National Health Service (NHS) spine portal on 17^th^ May 2023.

The study complies with the Declaration of Helsinki and was approved by the East of England—Cambridge Central Research Ethics Committee (REC reference 21/EE/0037) who waived the necessity of written consent according to the nature of the study (a register-based study with de-identified data and no active participation by study subjects).

### Cardiovascular magnetic resonance protocol

Cardiovascular magnetic resonance was performed on a 1.5 T CMR scanner (Aera, Siemens Healthineers, Erlangen, and Germany). The CMR protocol and sequences have been described previously [4]. In summary, we acquired: (i) conventional cine imaging; (ii) mid-short axis native T1 and T2 maps; and (iii) where indicated, late gadolinium enhancement (LGE) imaging.

All CMR studies were reported by experienced clinical CMR observers blinded to patient outcomes and analysed using “in-house” plug-ins for OsiriX MD version 9.0.1 (Pixmeo Sarl, Bernex, and Switzerland) as previously described [8]. Biventricular volumes (end-diastolic volume [EDV], end-systolic volume [ESV], stroke volume [SV]), ejection fractions (EF) and myocardial masses were calculated as previously described [9] with papillary muscles and trabeculae excluded from the blood pool. Bi-atrial areas were traced on a 4-chamber cine image at end-systole. All cardiac volumes, areas and masses were indexed (i) to body surface area. Native myocardial T1 and T2 relaxation times were measured by drawing a single region of interest (ROI) within the interventricular septum on the mid-cavity short-axis maps remote from areas of LGE and the insertion points [10].

Right and left ventricular (RV and LV) volumes were used to classify patients to one of five CMR-based phenotypes (CMR-SSc) recently demonstrated to have prognostic significance [4]. Patients were also identified as having primary cardiomyopathy of SSc (PC-SSc) by abnormal LVEF and/or LGE without other causes [11].

### Statistics

All statistical testing was performed using R (RStudio 2021.09.02 using R 4.1.2). As most descriptive variables were non-normally distributed, groups are described by median and full range with comparisons evaluated using Wilcoxon rank sum tests. Univariable survival analysis was performed using Cox Proportional Hazards (CPH) regression with hazard ratio (HR) reported per standard deviation change in continuous metrics. The most predictive variables (by HR) plus age were inputted into a multiple CPH model, including adjustments for: (1) CMR-SSc group [4]; (2) presence of PC-SSc [11], and (3) PH subtype. Optimal cut-offs for prognostic metrics were identified using maximally selected rank statistics and used to construct Kaplan-Meier plots and perform both omnibus and pairwise Log-Rank test (with adjustment for multiple comparisons performed using the Benjamini and Hochberg method). For all tests, a *P-value* <0.05 was considered statistically significant.

## Results

### Study population

The median age was 63 [range 21–85] years and patients were predominantly female (86%). The majority of PH diagnoses in our population were in group 1 (69%), with 20% in group 2, 10% in group 3 and 1% in group 4. Full SSc descriptors and targeted PH therapies are shown in Table 1.

**Table 1:** Clinical details of the study cohort

	SSc-PH patients (n = 148)
**Demographics and co-morbidities**	
Female, n (%)	127 (86)
Age (median [range]), years	63 (21–85)
BSA (median [range]), m^2^	1.69 (1.21–2.35)
Interstitial lung disease (any severity), n (%)	66 (45)
Emphysema (any severity), n (%)	27 (18)
Hypertension, n (%)	43 (29)
Diabetes mellitus, n (%)	14 (10)
Ischaemic heart disease, n (%)	12 (8)
Atrial fibrillation, n (%)	16 (11)
**Immunomodulatory therapy**	
Mycophenolate mofetil, n (%)	51 (35)
Glucocorticoids, n (%)	45 (30)
Azathioprine, n (%)	1 (0.7)
Hydroxychloroquine, n (%)	35 (24)
Leflunomide, n (%)	2 (1)
Tacrolimus, n (%)	2 (1)
Rituximab, n (%)	11 (7)
Cyclophosphamide, n (%)	13 (9)
Nintedanib, n (%)	1 (0.7)
Tocilizumab, n (%)	1 (0.7)
**SSc details**	
Limited/diffuse/sine SSc, n (%)	111/35/2 (75/24/1)
Overlap syndromes, n (%)	45 (30)
- Myositis, n (%)	23 (16)
- Inflammatory arthritis, n (%)	11 (7)
- Sjögren’s syndrome, n (%)	13 (9)
- SLE, n (%)	9 (6)
SSc disease duration (from SSc diagnosis to CMR, median [range]), years	12 (0–42)
SSc disease severity score (median [range])	3 (0–4)
**Autoantibody subset, %**	
Anticentromere	70 (47)
Anti-topoisomerase I	17 (12)
Anti-RNA polymerase	11 (7)
Anti-U3 RNP	10 (7)
Anti-PM/Scl	2 (1)
Anti-U1 RNP	17 (12)
Anti-Th/To	5 (3)
ANA+ENA-	68 (46)
ANA-	4 (3)
**SSc-PH details**	
group 1/2/3/4 PH, n (% of PH diagnoses)	102/29/15/2 (69/20/10/1)
Incident/Prevalent group 1 PH diagnosis, n (%)	32/70 (31/69)
SSc-PH disease duration for prevalent patients (from SSc-PH diagnosis to CMR, median [range]), years	2.1 (0.1–16.8)
**Targeted therapies for group 1 PH patients (*n* = 102)**	
PDE5I, n (% of group 1 PH patients)	68 (67)
ERA, n (% of group 1 PH patients)	50 (49)
SGCS, n (% of group 1 PH patients)	4 (4)
Prostanoids (IV/oral), n (% of group 1 PH patients)	4 (4)/2 (2)

Values are median (range) or n (%).

ANA: antinuclear antibody; BSA: body surface area; CMR: cardiovascular magnetic resonance; ENA: anti-extractable nuclear antigen; ERA: endothelin receptor antagonist; IV: intravenous; m: metres; PDE5I: phosphodiesterase type 5 inhibitor; PH: pulmonary hypertension; SGCS: soluble guanylate cyclase stimulator; SLE: systemic lupus erythematosus; SSc: scleroderma/systemic sclerosis.

### Predictors of mortality

Sixty-six (45%) patients died during a median follow-up period of 3.5 years (range 0.1–7.3 years). One-year, three-year and five-year survival was 87%, 65%, and 42% respectively. Patients who died during follow-up (Supplementary Table S1, available at *Rheumatology* online) were older (*P* = 0.035) with more dilated (*P* < 0.001), hypertrophied (*P* = 0.013) and impaired (*P* < 0.001) right ventricles, larger right atria (*P* = 0.043) and higher native T1 (*P* < 0.001), but not higher T2 (*P* = 0.33).

Supplementary Fig. S1, available at *Rheumatology* online shows univariable CMR predictors of mortality. Indexed RV end-systolic volume (RVESVi), RV mass (RVMi), right atrial area (RAi), LV stroke volume (LVSVi) and native T1 were entered into a multiple CPH model. After adjustment for age, only RVESVi (*P* = 0.0023) and native T1 (*P* = 0.0024) were independent predictors of all-cause mortality. Both variables remained independent predictors after adjusting for: (i) CMR-SSc phenotype (RVESVi *P* = 0.026, T1 *P* = 0.0021) and (ii) presence of PC-SSc (RVESVi *P* < 0.001, T1 *P* = 0.0023), (iii) PH subtype (RVESVi *P* < 0.001, T1 *P* = 0.0056).

### Defining thresholds

**Figure 1.**
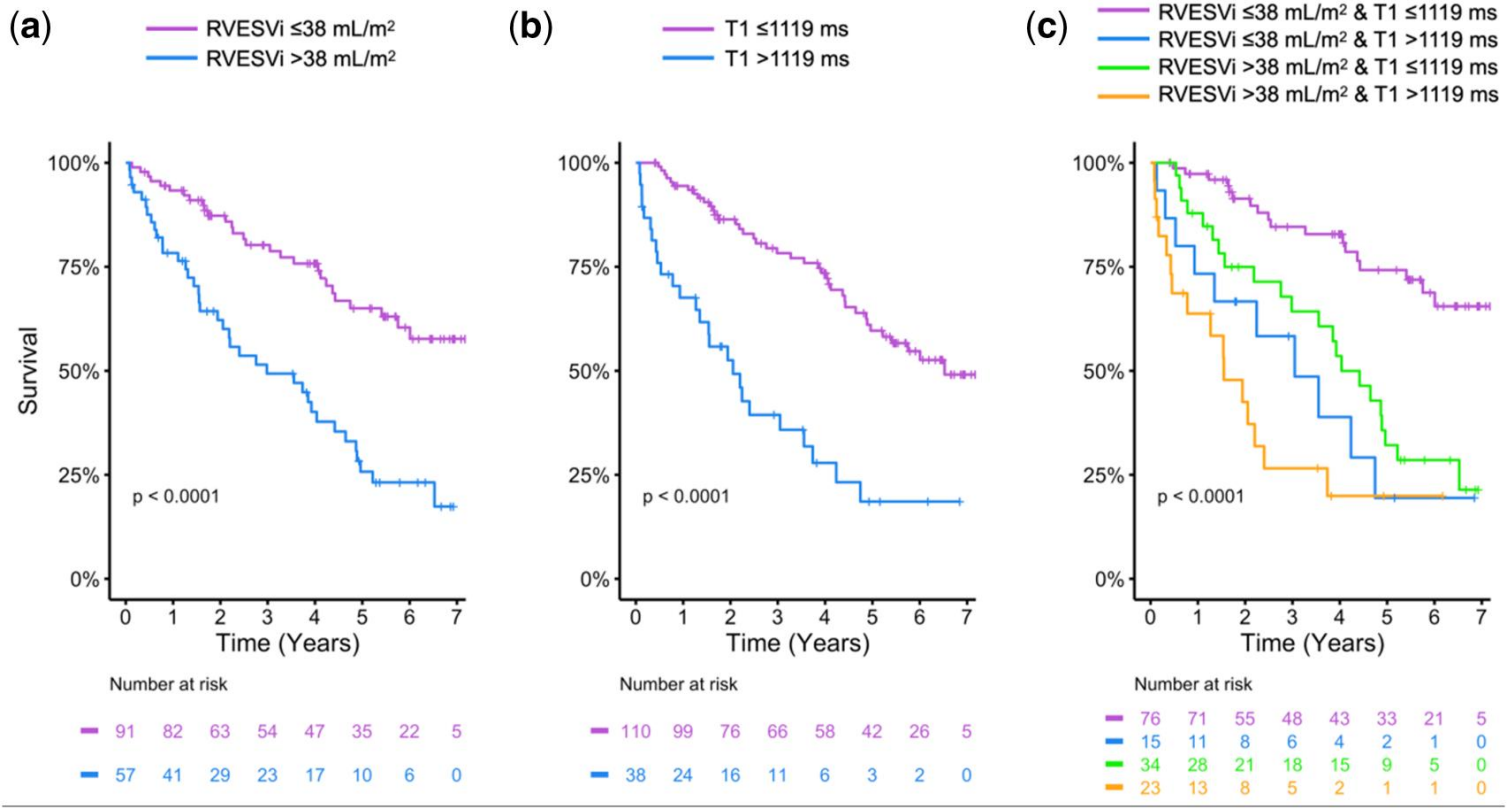
Kaplan-Meier curves for: (a) RVESVi and (b) native myocardial T1, with optimal thresholds to predict adverse prognosis of RVESVi >38mL/m^2^ and T1 > 1119ms (*P* < 0.001); (c) all combinations (ie high or low) of RVESVi and native myocardial T1

**Figure 2.**
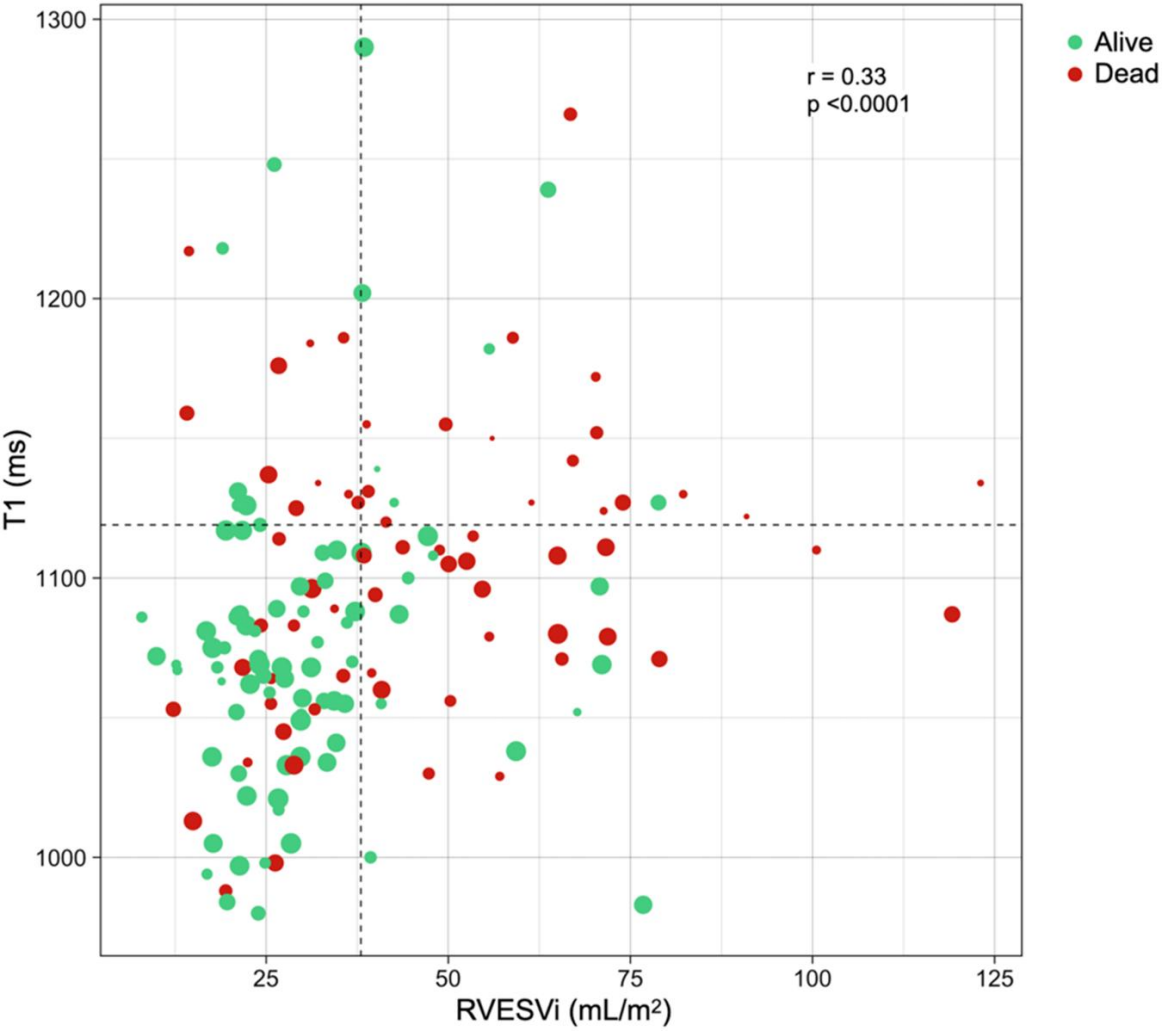
Scatter plot of patients by RVESVi and native myocardial T1. Patients are classified as alive (green) or dead (red) at the time of censorship, with the sizes of data points proportional to the time of event (death) or censorship (alive)

The optimal thresholds of RVESVi and native T1 to predict adverse prognosis were RVESVi >38mL/m^2^ and T1 > 1119ms (*P* < 0.001, Fig. 1). Kaplan-Meier curves for all combinations (ie high or low) of these two variables (Fig. 1) showed that patients with RVESVi ≤38 mL/m^2^ and native T1 ≤ 1119 ms had significantly better outcomes than all three other groups (*P* < 0.001). Furthermore, patients with RVESVi >38mL/m^2^ and native T1 ≤ 1119 ms had significantly better survival than patients with RVESVi >38mL/m^2^ and native T1 > 1119ms (*P* = 0.017). This can also be seen in Fig. 2, whereby patients with higher RVESVi and/or native T1 were more likely to be dead with a shorter time to event (or censorship if alive).

## Discussion

In a large cohort of patients with SSc-PH, we have demonstrated that RVESVi and native myocardial T1 by CMR are independent predictors of all-cause mortality. These metrics are prognostic even when adjusted for PC-SSc, PH subtype, and CMR-derived SSc phenotype, demonstrating their additional clinical utility. Importantly, we have developed thresholds based on maximally selected rank statistics to aid clinical use. We showed that cut-offs of RVESVi >38 ml/m^2^ and native T1 > 1119ms optimally separated patients with a poorer prognosis, and patients with both had the poorest outcomes and more imminently.

The response of the RV to elevated afterload is known to be the major determinant of outcome in PH [12]. Previous CMR studies have shown that RVESVi independently predicts outcome in patients with group 1 PH due to multiple aetiologies, which is comparable to our findings in patients with SSc-PH [13–15]. Furthermore, a threshold of RVESVi ≤41.75 mL/m^2^ was shown to be associated with a low-risk status in patients with group 1 PH [14]. This is close to our optimal prognostic cut-off for RVESVi in SSc-PH of ≤38 mL/m^2^, with methodological differences in cavity segmentation and our comparatively older study population being potential contributors to the slightly lower RVESVi [16]. Overall, these findings reinforce the position of CMR as the preferred imaging modality for accurate and reproducible RV volumetric quantification.

While RV volumes are recognized predictors of outcome in all forms of PH, the prognostic value of native myocardial T1 in PH is less well established. A meta-analysis of patients with groups 1 and 4 PH reported significantly higher native myocardial T1 compared with controls, particularly at the RV insertion points, likely due to afterload-related diffuse fibrosis [17]. Furthermore, T1 values were associated with larger and poorer right ventricles. Despite this association, a study of patients with group 1 PH of mixed aetiologies found no association between native T1 and mortality [18]. Our study, however, shows that native T1 is prognostic in SSc-PH independently of right heart size and function. We have also previously shown that native myocardial T1 is prognostic in SSc independently of the presence of a PH diagnosis [4]. These findings suggest that elevated native T1 in SSc-PH is, at least in part, representative of myocardial disease due to SSc itself, rather than being solely due to the effects of PH. This may also be relevant to management strategies, with recent international guidelines recommending treatment of both the underlying connective tissue disease as well as any associated PH [19].

Myocardial T1 is elevated in the presence of water, both when present as free water or when bound to larger molecules such as collagen [20]. Hence elevated native T1 in SSc might represent myocardial oedema due to inflammation, diffuse myocardial fibrosis or a combination of both. Myocardial T2, however, is more specific for oedema and was not prognostic in this cohort of patients. This suggests that myocardial inflammation is less contributory to outcome in SSc-PH compared with other cardiovascular phenotypes in SSc that exhibit characteristics suggestive of a pro-inflammatory state [4]. Further work should be directed at assessing if native T1 is affected by immunomodulatory or antifibrotic therapies and hence if it could be used as a treatment target in patients with SSc, with or without PH.

### Limitations

To the best of our knowledge, this study of CMR metrics in SSc-PH is the largest to date in this rare disease. Nevertheless, this is a single-site, retrospective study of patients with SSc-PH, the majority of whom were prevalent for their diagnosis. As such, conclusions from this study will be prone to survivorship bias with no way of knowing the CMR characteristics of those patients who died without undergoing a CMR study. Validation of our findings, including the thresholds for RVESVi and native T1, is required in prospective multicentre patient cohorts. Contemporaneous risk stratification metrics used to assess disease severity in PH, such as brain natriuretic peptide (BNP) levels and 6-min walk distance, along with other non-CMR parameters, including pulmonary function tests and invasive haemodynamics, were not available in all patients. Future work using a prospective study design with broader inclusion of contemporaneously acquired multi-modality data would enable the incremental value of CMR metrics in SSc-PH to be investigated alongside non-CMR metrics and PH risk markers. Finally, we pooled all PH subtypes in our analysis and lack statistical power for sub-group analysis. However, it should be noted that RVESVi and T1 remained prognostic even after adjustment for the PH subtype.

## Conclusion

Both RVESVi and native myocardial T1 are independently predictive of mortality in SSc-PH. In these patients, native myocardial T1 may reflect a composite effect of SSc cardiac involvement and the effects of PH on the myocardium. Assessing myocardial tissue characterization alongside RV size and function confers added value in SSc-PH and could be useful in tracking disease progression and response to therapy in these patients.

## Supplementary Material

keae141_Supplementary_Data

## Data Availability

All data underlying this article are included in the article and its online supplementary material. The data that support this study are available on a reasonable request to the corresponding author, subject to institutional and ethical committee approvals.

## References

[1] Haque A , Kiely DG, Kovacs G, Thompson AAR, Condliffe R. Pulmonary hypertension phenotypes in patients with systemic sclerosis. Eur Respir Rev 2021;30:210053. 34407977 10.1183/16000617.0053-2021 PMC9517999

[2] Swift AJ , Rajaram S, Campbell MJ et al Prognostic value of cardiovascular magnetic resonance imaging measurements corrected for age and sex in idiopathic pulmonary arterial hypertension. Circ Cardiovasc Imaging 2014;7:100–6. 24275955 10.1161/CIRCIMAGING.113.000338

[3] van Wolferen SA , Marcus JT, Boonstra A et al Prognostic value of right ventricular mass, volume, and function in idiopathic pulmonary arterial hypertension. Eur Heart J 2007;28:1250–7. 17242010 10.1093/eurheartj/ehl477

[4] Knight DS , Karia N, Cole AR et al Distinct cardiovascular phenotypes are associated with prognosis in systemic sclerosis: a cardiovascular magnetic resonance study. Eur Heart J Cardiovasc Imaging 2023;24:463–71. 35775814 10.1093/ehjci/jeac120 PMC10029850

[5] Galie N , Humbert M, Vachiery JL et al; ESC Scientific Document Group. 2015 ESC/ERS Guidelines for the diagnosis and treatment of pulmonary hypertension: the Joint Task Force for the Diagnosis and Treatment of Pulmonary Hypertension of the European Society of Cardiology (ESC) and the European Respiratory Society (ERS): Endorsed by: Association for European Paediatric and Congenital Cardiology (AEPC), International Society for Heart and Lung Transplantation (ISHLT). Eur Heart J 2016;37:67–119. 26320113 10.1093/eurheartj/ehv317

[6] van den Hoogen F , Khanna D, Fransen J et al 2013 classification criteria for systemic sclerosis: an American College of Rheumatology/European League against Rheumatism collaborative initiative. Arthritis Rheum 2013;65:2737–47. 24122180 10.1002/art.38098 PMC3930146

[7] Medsger TA Jr , Silman AJ, Steen VD et al A disease severity scale for systemic sclerosis: development and testing. J Rheumatol 1999;26:2159–67. 10529133

[8] Rosset A , Spadola L, Ratib O. OsiriX: an open-source software for navigating in multidimensional DICOM images. J Digit Imaging 2004;17:205–16. 15534753 10.1007/s10278-004-1014-6 PMC3046608

[9] Knight DS , Zumbo G, Barcella W et al Cardiac structural and functional consequences of amyloid deposition by cardiac magnetic resonance and echocardiography and their prognostic roles. JACC Cardiovasc Imaging 2019;12:823–33. 29680336 10.1016/j.jcmg.2018.02.016

[10] Messroghli DR , Moon JC, Ferreira VM et al Clinical recommendations for cardiovascular magnetic resonance mapping of T1, T2, T2 and extracellular volume: a consensus statement by the Society for Cardiovascular Magnetic Resonance (SCMR) endorsed by the European Association for Cardiovascular Imaging (EACVI). J Cardiovasc Magn Reson 2017;19:75. 28992817 10.1186/s12968-017-0389-8 PMC5633041

[11] Chhikara S , Kanda A, Ogugua FM et al The primary cardiomyopathy of systemic sclerosis on cardiovascular magnetic resonance imaging. Eur Heart J Cardiovasc Imaging 2023;24:1661–71. 37364296 10.1093/ehjci/jead147

[12] Chin KM , Kim NH, Rubin LJ. The right ventricle in pulmonary hypertension. Coron Artery Dis 2005;16:13–8. 15654194 10.1097/00019501-200502000-00003

[13] Alabed S , Shahin Y, Garg P et al Cardiac-MRI predicts clinical worsening and mortality in pulmonary arterial hypertension: a systematic review and meta-analysis. JACC Cardiovasc Imaging 2021;14:931–42. 33008758 10.1016/j.jcmg.2020.08.013 PMC7525356

[14] Lewis RA , Johns CS, Cogliano M et al Identification of cardiac magnetic resonance imaging thresholds for risk stratification in pulmonary arterial hypertension. Am J Respir Crit Care Med 2020;201:458–68. 31647310 10.1164/rccm.201909-1771OC PMC7049935

[15] Swift AJ , Capener D, Johns C et al Magnetic resonance imaging in the prognostic evaluation of patients with pulmonary arterial hypertension. Am J Respir Crit Care Med 2017;196:228–39. 28328237 10.1164/rccm.201611-2365OC PMC5519970

[16] Foppa M , Arora G, Gona P et al Right ventricular volumes and systolic function by cardiac magnetic resonance and the impact of sex, age, and obesity in a longitudinally followed cohort free of pulmonary and cardiovascular disease: the Framingham Heart Study. Circ Cardiovasc Imaging 2016;9:e003810. 26962126 10.1161/CIRCIMAGING.115.003810

[17] Alabed S , Saunders L, Garg P et al Myocardial T1-mapping and extracellular volume in pulmonary arterial hypertension: a systematic review and meta-analysis. Magn Reson Imaging 2021;79:66–75. 33745961 10.1016/j.mri.2021.03.011

[18] Saunders LC , Johns CS, Stewart NJ et al Diagnostic and prognostic significance of cardiovascular magnetic resonance native myocardial T1 mapping in patients with pulmonary hypertension. J Cardiovasc Magn Reson 2018;20:78. 30501639 10.1186/s12968-018-0501-8 PMC6276188

[19] Humbert M , Kovacs G, Hoeper MM et al; ESC/ERS Scientific Document Group. 2022 ESC/ERS Guidelines for the diagnosis and treatment of pulmonary hypertension. Eur Heart J 2022;43:3618–731. 36017548 10.1093/eurheartj/ehac237

[20] Maestrini V , Treibel TA, White SK, Fontana M, Moon JC. T1 mapping for characterization of intracellular and extracellular myocardial diseases in heart failure. Curr Cardiovasc Imaging Rep 2014;7:9287. 25152807 10.1007/s12410-014-9287-8 PMC4133016

