# Supplementary material for: Native myocardial T1 and right ventricular size by CMR predict outcome in systemic sclerosis-associated pulmonary hypertension
Source: Rheumatology (Oxford). 2024 May 17;63(10):2678–83. doi: 10.1093/rheumatology/keae141 (PMC11443025; doi:10.1093/rheumatology/keae141)
Supplement: keae141_Supplementary_Data [file keae141_supplementary_data.docx]

**Supplementary material**

Knight DS, Virsinskaite R, Karia N, et al. Native myocardial T1 and right ventricular size by cardiovascular magnetic resonance predict outcome in systemic sclerosis-associated pulmonary hypertension.

**Appendix**

- **Supplementary** **Table S1.** Clinical and CMR parameters of patients according to survival at follow-up.
- **Supplementary Figure S1.** Forest plot of CMR variables to predict all-cause mortality on univariable Cox analysis.

This supplemental material has been provided by the authors to give readers additional information about their work.

**Supplementary** **Table S1:** Clinical and CMR parameters of patients according to survival at follow-up.

|  | **Total cohort (n = 148)** | **Alive (n = 82)** | **Dead (n = 66)** | ***P* value** |
| --- | --- | --- | --- | --- |
| **CMR metrics** | | | | |
| RVEDVi (mL/m^2^) | 78 (30-184) | 69 (30-125) | 84 (41-184) | **<0.001** |
| RVESVi (mL/m^2^) | 32 (8-123) | 27 (8-79) | 40 (12-123) | **<0.001** |
| RVSVi (mL/m^2^) | 42 (18-77) | 42 (18-72) | 42 (21-77) | 0.80 |
| RVEF (%) | 57 (19-82) | 61 (20-80) | 51 (19-82) | **<0.001** |
| RVMi (g/m^2^) | 33 (14-86) | 30 (14-66) | 39 (18-86) | **0.013** |
| LVEDVi (mL/m^2^) | 64 (27-131) | 65 (27-117) | 62 (36-131) | 0.49 |
| LVESVi (mL/m^2^) | 19 (6-82) | 20 (6-76) | 19 (6-82) | 0.74 |
| LVSVi (mL/m^2^) | 42 (17-71) | 43 (17-71) | 41 (19-66) | 0.060 |
| LVEF (%) | 67 (28-86) | 68 (33-84) | 66 (28-86) | 0.20 |
| LVMi (g/m^2^) | 63 (29-140) | 63 (43-127) | 65 (29-140) | 0.95 |
| LAi area (cm/m^2^) | 13 (5-21) | 13 (5-21) | 13 (6-21) | 0.75 |
| RAi area (cm/m^2^) | 13 (6-22) | 12 (6-22) | 14 (7-22) | **0.043** |
| T1 (ms) | 1084 (980-1290) | 1071 (980-1290) | 1108 (988-1266) | **<0.001** |
| T2 (ms) | 51 (40-64) | 50 (41-64) | 51 (40-60) | 0.33 |
| Pericardial effusion, n (%) | 55/148 (37) | 29/82 (35) | 26/66 (39) | 0.73 |
| Any major LGE*, n (%) | 21/123 (17) | 11/66 (17) | 10/57 (18) | >0.99 |
| Widespread subendocardial/trabecular LGE, n (%) | 12/123 (10) | 5/66 (8) | 7/57 (12) | 0.54 |
| Midwall LGE, n (%) | 17/123 (14) | 10/66 (15) | 7/57 (12) | 0.79 |
| Meeting definition of primary cardiomyopathy of SSc, n (%) | 24/148 (16) | 9/82 (11) | 15/66 (23) | 0.072 |
| **Clinical metrics** | | | | |
| Male, n (%) | 21/148 (14) | 8/82 (10) | 13/66 (20) | 0.10 |
| Age, years | 63 (21-85) | 60 (25-78) | 65 (21-85) | **0.035** |
| SSc disease duration (from SSc diagnosis to CMR), years | 12 (0-42) | 12 (1-36) | 12 (0-42) | 0.060 |
| SSc disease severity score | 3 (0-4) | 2 (1-4) | 3 (0-4) | **<0.001** |
| SSc-PH disease duration for prevalent patients (from SSc-PH diagnosis to CMR, median [range]), years | 2.1 (0.1-16.8) | 2.6 (0.1-16.8) | 1.7 (0.1-13.9) | 0.30 |
| Heart rate (beats per minute) | 75 (48-113) | 75 (48-109) | 76 (49-113) | 0.41 |

Values are median (range) or n (%).

Statistically significant metrics (*P* <0.05) are denoted by *P* values in bold text.

cm, centimetres; CMR, cardiovascular magnetic resonance; EDV, end-diastolic volume; EF, ejection fraction; ESV, end-systolic volume; g, grammes; i, indexed for body-surface area; LA, left atrial; LGE, late gadolinium enhancement; LV, left ventricular; M, mass; m, metres; mL, millilitres; ms, milliseconds; RA, right atrial; RV, right ventricular; SSc, scleroderma/systemic sclerosis; SV, stroke volume.

*excluding minor insertion point LGE. 123 patients received gadolinium-based contrast.

**Supplementary Figure S1.** Forest plot of CMR variables to predict all-cause mortality on univariable Cox analysis.


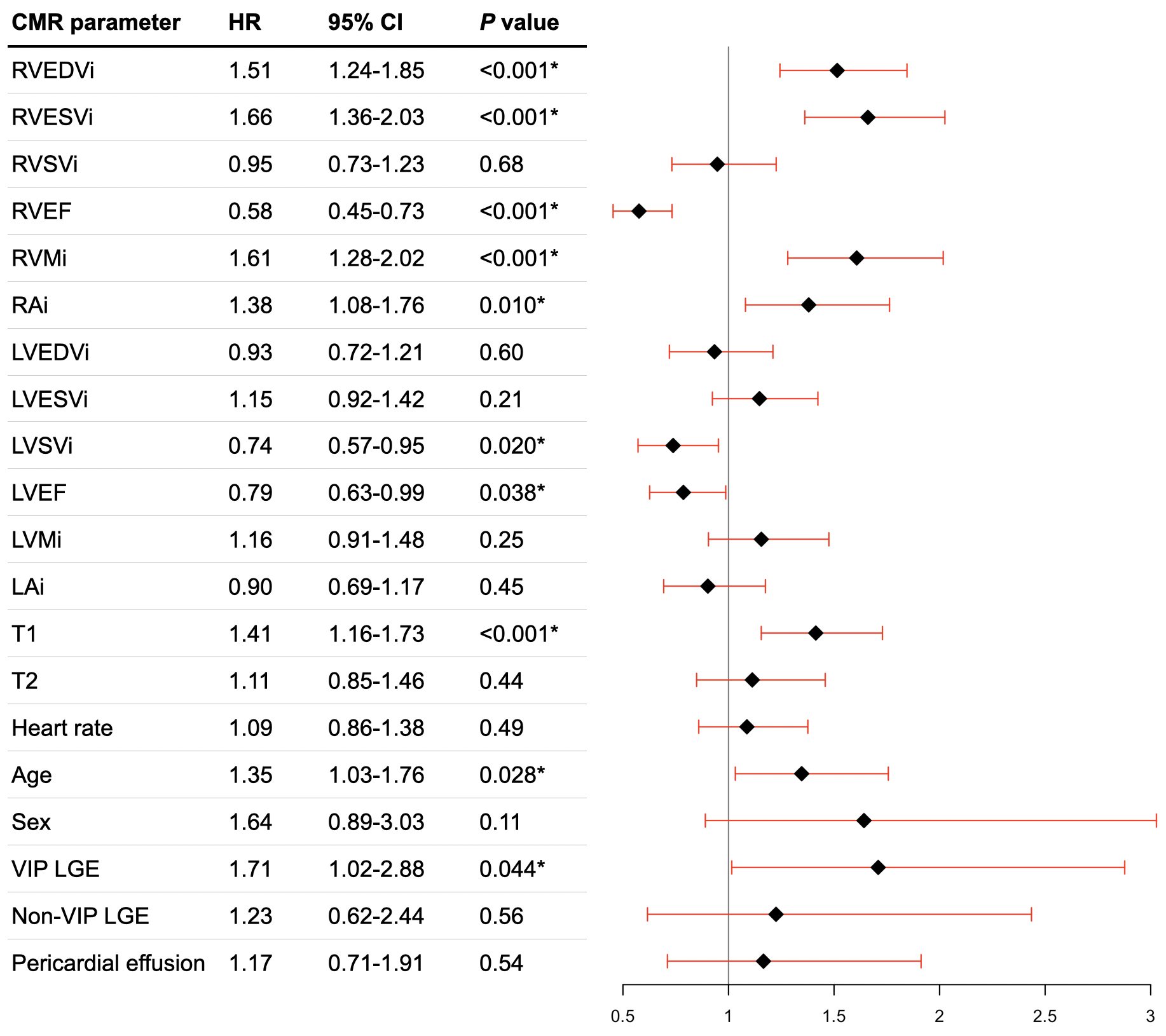


*denotes statistically significant metrics (*P* <0.05).

CI, confidence intervals; CMR, cardiovascular magnetic resonance; EDV, end-diastolic volume; EF, ejection fraction; ESV, end-systolic volume; HR, hazard ratio; i, indexed for body-surface area; LA, left atrial area; LGE, late gadolinium enhancement; LV, left ventricular; M, mass; RA, right atrial area; RV, right ventricular; SV, stroke volume; VIP, ventricular insertion point.
